# Crystal structure of 5-(4-benzyl­oxyphen­yl)-3-(4-meth­oxy­phen­yl)-6-methyl­cyclo­hex-2-en-1-one

**DOI:** 10.1107/S2056989014025390

**Published:** 2015-01-01

**Authors:** S. Sathya, D. Reuben Jonathan, J. Sidharthan, R. Vasanthi, G. Usha

**Affiliations:** a PG and Research Department of Physics, Queen Mary’s College, Chennai-4, Tamilnadu, India; b Department of Chemistry, Madras Christian College, Chennai-59, Tamilnadu, India; c PG & Research Department of Chemistry, V. O. Chidambaram College, Thoothukudi - 628 008, Tamil Nadu, India

**Keywords:** crystal structure, cyclo­hexene, cyclcohexen-1-one

## Abstract

The title compound, C_27_H_26_O_3_, crystallized with two independent mol­ecules (*A* and *B*) in the asymmetric unit. In mol­ecule *A*, the plane of the central benzene ring forms dihedral angles of 75.78 (14) and 52.75 (16)° with that of the terminal benzene rings, and the dihedral angle between the planes of the terminal benzene rings is 51.49 (17)°. The corresponding values for mol­ecule *B* are 75.18 (14), 58.11 (16) and 47.91 (16)°, respectively. The cyclo­hexene ring adopts an envelope conformation in both mol­ecules, with the C atom to which is attached the central benzene ring as the flap. The crystal packing, is stabilized by C—H⋯π inter­actions.

## Abstract 2

5-(4-Benzyl­oxyphen­yl)-3-(4-meth­oxy­phen­yl)-6-methyl­cyclo­hex-2-en-1-one crystallized with two independent mol­ecules in the asymmetric unit. The cyclo­hexene ring adopts an envelope conformation in both mol­ecules, with the C atom to which is attached the central benzene ring as the flap. The crystal packing, is stabilized by C—H⋯π inter­actions.

## Structural commentary   

Cyclo­hexenone is a versatile inter­mediate used in the synthesis of a variety of chemical products such as pharmaceuticals and fragrances. Cyclo­hexenone and cyclo­hexenone derivatives are known for anti-inflammatory and analgesic activities (Kalluraya & Rahiman, 2003 ▸). α,β-Unsaturated carbonyl compounds have shown various biological activities such as anti­oxidant (Suksamrarn *et al.*, 2003 ▸), anti­tumor (Kumar *et al.*, 2003 ▸), anti­cancer (Modzelewska *et al.*, 2006 ▸) and anti­malarial (Ferrer *et al.*, 2009 ▸). In addition, chalcones are widely used in cosmetic compositions (Forestier *et al.*, 1989 ▸; Podraze, 1991 ▸) and in applications of dyes (Asiri, 2003 ▸). Cyclo­hexenone derivatives are well known lead mol­ecules for the treatment of inflammation and autoimmune diseases (Tanaka *et al.*, 1997 ▸). Apart from being biologically important compounds, chalcone derivatives show nonlinear optical (NLO) properties with excellent blue light transmittance and good crystallizability (Shettigar *et al.*, 2006 ▸). In this context, herein we report the synthesis and crystal structure of the title compound.
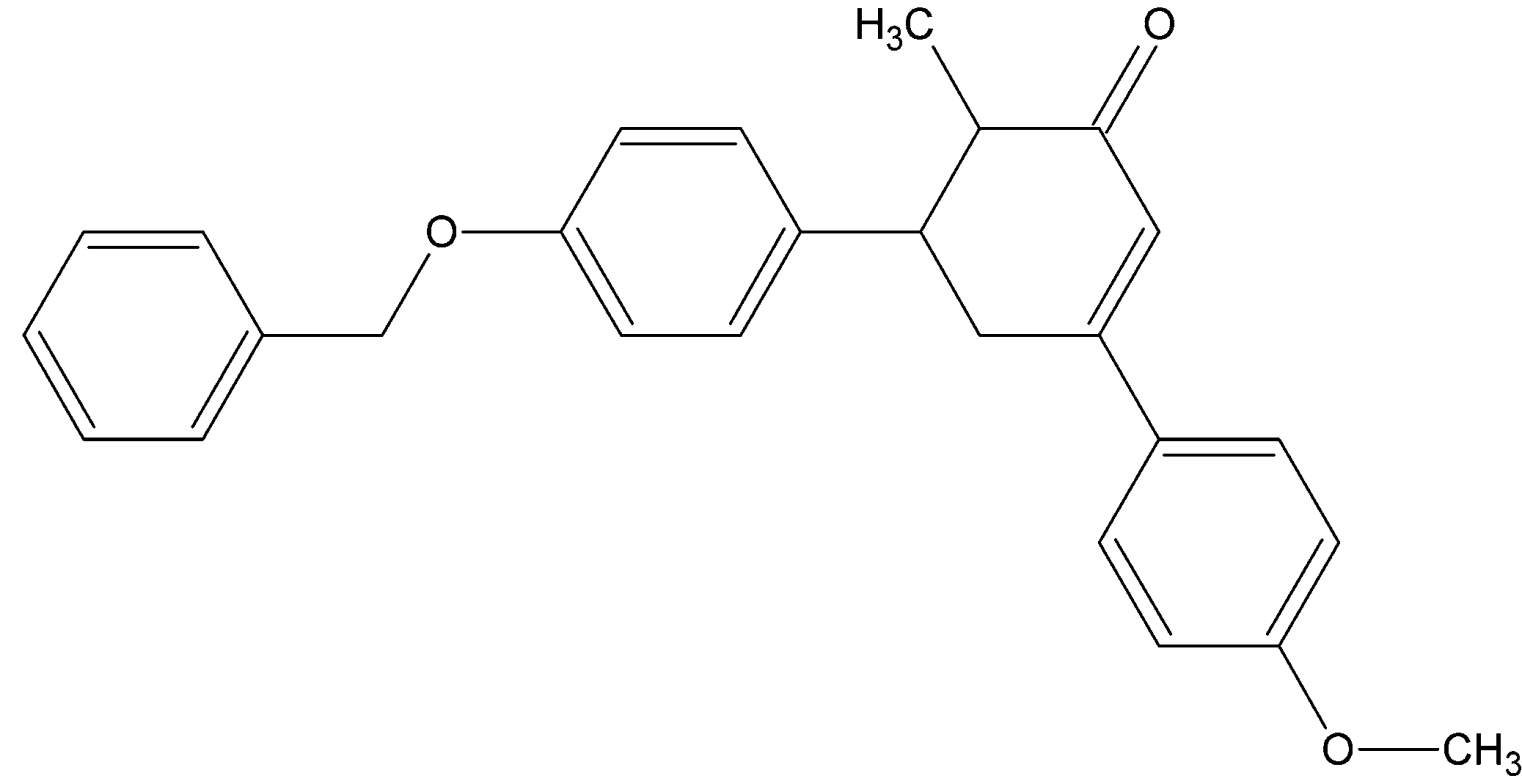


**Figure 1:**
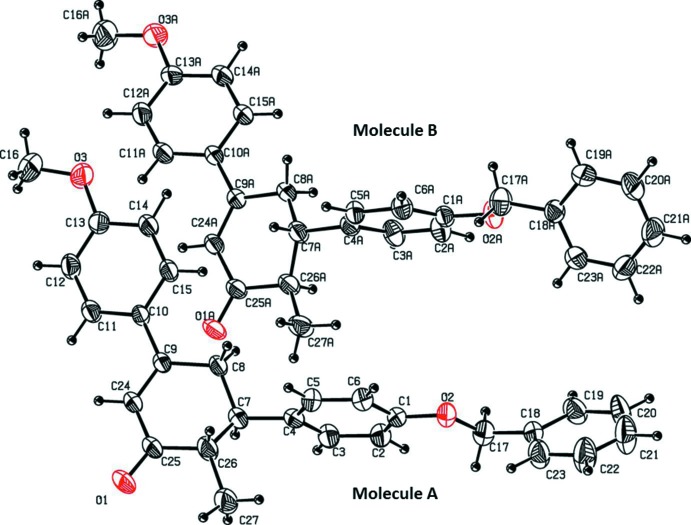
The mol­ecular structure of the two independent mol­ecules of the title compound, showing the atom labelling. Displacement ellipsoids are drawn at the 30% probability level.

The title compound crystallized with two mol­ecules (*A* and *B*) in the asymmetric unit (Fig. 1 ▸). The benzyl­oxyphenyl and the meth­oxy­phenyl rings are linked with a cyclo­hexene ring. The C25—O1 and C25*A*—O1*A* bond lengths of 1.228 (3) and 1.224 (3) Å, respectively, indicate double-bond character. In both mol­ecules, the C—O bond lengths are in the range 1.362 (3)–1.414 (4) Å and represent single-bond character. In mol­ecule *A*, the torsion angles C5—C4—C7—C8 = 69.2 (4)° and C24—C9—C10—C11 = −16.0 (4)° show that the benzyl­oxyphenyl and meth­oxy­phenyl groups have a +*sc* and -*sp* orientation with respect to the cyclo­hexene moiety. The arrangement in mol­ecule *B* is slightly different, with torsion angles C5*A*—C4*A*—C7*A*—C8*A* = 111.5 (3)° and C24*A*—C9*A*—C10*A*—C11*A* = 20.8 (4)°. The cyclo­hexene ring in both mol­ecules adopts an envelope conformation with atoms C7 and C7*A* as the flaps in mol­ecules *A* and *B*, respectively.

**Figure 2:**
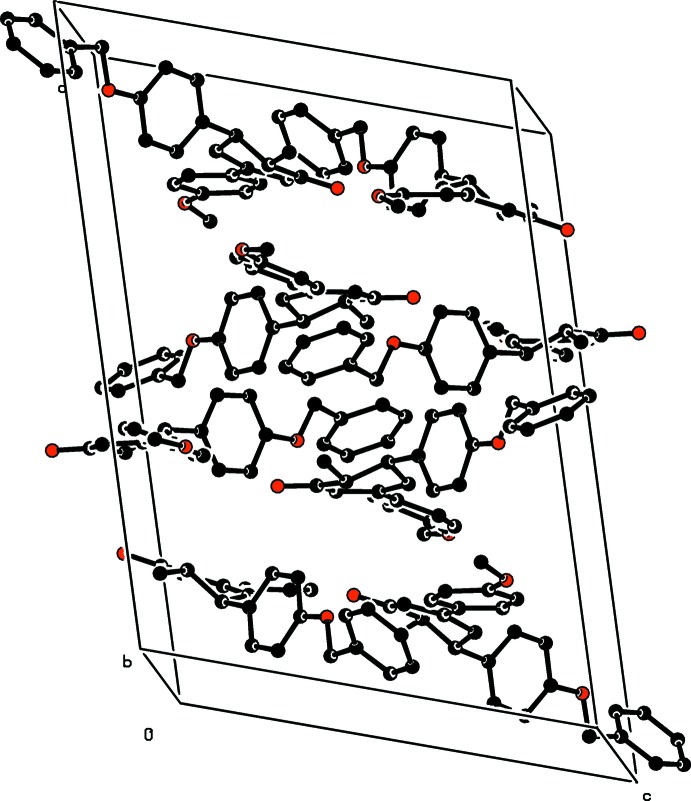
A view along the *b* axis of the crystal packing of the title compound.

The crystal packing (Fig. 2 ▸) is stabilized by C—H⋯π inter­actions (Table 1 ▸).

## Synthesis and crystallization   

(2*E*)-3-(4-Benzyl­oxyphen­yl)-1-(4-meth­oxy­phen­yl)prop-2-en-1-one was synthesized following the literature procedure of Ezhilarasi *et al.* (2014 ▸). The synthesis of the title compound was carried out by following the reported procedure of Fun *et al.* (2012 ▸). In a 100 ml round-bottomeded flask, (2*E*)-3-(4-benzyl­oxyphen­yl)-1-(4-meth­oxy­phen­yl)prop-2-en-1-one (0.01 mol) and ethyl methyl ketone (0.01 mol) were refluxed in absolute alcohol (50 ml) in the presence of 10% sodium hydroxide solution (10 ml) for 1 h in an oil bath. The reaction mixture was then cooled and the precipitate obtained filtered, washed with distilled water and dried. The crude product was recrystallized twice from absolute alcohol (yield 80%), giving yellow block-like crystals.

## Refinement   

Crystal data, data collection and structure refinement details are summarized in Table 2 ▸. The H atoms were positioned geometrically and treated as riding atoms, with C—H = 0.93–0.98 Å, and with *U*_iso_(H) = 1.5*U*_eq_(C) for methyl H atoms and 1.2*U*_eq_(C) for other H atoms.

## Supplementary Material

Crystal structure: contains datablock(s) I, New_Global_Publ_Block. DOI: 10.1107/S2056989014025390/su5016sup1.cif

Structure factors: contains datablock(s) I. DOI: 10.1107/S2056989014025390/su5016Isup2.hkl

Supporting information file. DOI: 10.1107/S2056989014025390/su5016Isup3.cml

CCDC reference: 1035112

Additional supporting information:  crystallographic information; 3D view; checkCIF report

## Figures and Tables

**Table 1:** Hydrogen-bond geometry (, ) *Cg*3 and *Cg*8 are the centroids of rings C10-C15 and C10*A*-C15*A*, respectively.

*D*H*A*	*D*H	H*A*	*D* *A*	*D*H*A*
C2*A*H2*A* *Cg*3^i^	0.93	2.87	3.707(3)	150
C16*A*H16*E* *Cg*8^ii^	0.96	2.92	3.866(5)	168

Symmetry codes: (i) 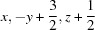; (ii) 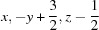.

**Table 2:** Experimental details

Crystal data	
Chemical formula	C_27_H_26_O_3_
*M*_r_	398.48
Crystal system, space group	Monoclinic, *P*2_1_/*c*
Temperature (K)	293
*a*, *b*, *c* ()	20.5663(12), 15.2878(9), 14.5689(8)
()	107.938(4)
*V* (^3^)	4358.0(4)
*Z*	8
Radiation type	Mo *K*
(mm^1^)	0.08
Crystal size (mm)	0.25 0.23 0.20

Data collection	
Diffractometer	Bruker Kappa APEXII CCD
Absorption correction	Multi-scan (*SADABS*; Bruker, 2008 ▸)
*T*_min_, *T*_max_	0.981, 0.985
No. of measured, independent and observed [*I* > 2(*I*)] reflections	33189, 7901, 3714
*R*_int_	0.057
(sin /)_max_ (^1^)	0.600

Refinement	
*R*[*F* ^2^ > 2(*F* ^2^)], *wR*(*F* ^2^), *S*	0.063, 0.193, 0.99
No. of reflections	7901
No. of parameters	546
H-atom treatment	H-atom parameters constrained
_max_, _min_ (e ^3^)	0.31, 0.21

Computer programs: *APEX2* and *SAINT* (Bruker, 2008 ▸), *SHELXS97* and *SHELXL2014* (Sheldrick, 2008 ▸), *ORTEP-3 for Windows* (Farrugia, 2012 ▸), and *PLATON* (Spek, 2009 ▸).

## supplementary crystallographic information

### Crystal data

**Table:**

C_27_H_26_O_3_	*F*(000) = 1696
*M**_r_* = 398.48	*D*_x_ = 1.215 Mg m^−^^3^
Monoclinic, *P*2_1_/*c*	Mo *K*α radiation, λ = 0.71073 Å
*a* = 20.5663 (12) Å	Cell parameters from 10904 reflections
*b* = 15.2878 (9) Å	θ = 1.0–28.4°
*c* = 14.5689 (8) Å	µ = 0.08 mm^−^^1^
β = 107.938 (4)°	*T* = 293 K
*V* = 4358.0 (4) Å^3^	Block, yellow
*Z* = 8	0.25 × 0.23 × 0.20 mm

### Data collection

**Table:**

Bruker Kappa APEXII CCD diffractometer	7901 independent reflections
Radiation source: fine-focus sealed tube	3714 reflections with *I* > 2σ(*I*)
Graphite monochromator	*R*_int_ = 0.057
ω and φ scan	θ_max_ = 25.3°, θ_min_ = 1.0°
Absorption correction: multi-scan (*SADABS*; Bruker, 2008)	*h* = −24→24
*T*_min_ = 0.981, *T*_max_ = 0.985	*k* = −18→14
33189 measured reflections	*l* = −17→17

### Refinement

**Table:**

Refinement on *F*^2^	Hydrogen site location: inferred from neighbouring sites
Least-squares matrix: full	H-atom parameters constrained
*R*[*F*^2^ > 2σ(*F*^2^)] = 0.063	*w* = 1/[σ^2^(*F*_o_^2^) + (0.1028*P*)^2^] where *P* = (*F*_o_^2^ + 2*F*_c_^2^)/3
*wR*(*F*^2^) = 0.193	(Δ/σ)_max_ < 0.001
*S* = 0.99	Δρ_max_ = 0.31 e Å^−^^3^
7901 reflections	Δρ_min_ = −0.21 e Å^−^^3^
546 parameters	Extinction correction: *SHELXL2014* (Sheldrick, 2008), Fc^*^=kFc[1+0.001xFc^2^λ^3^/sin(2θ)]^-1/4^
0 restraints	Extinction coefficient: 0.0020 (5)

### Special details

**Table:**

Geometry. All esds (except the esd in the dihedral angle between two l.s. planes) are estimated using the full covariance matrix. The cell esds are taken into account individually in the estimation of esds in distances, angles and torsion angles; correlations between esds in cell parameters are only used when they are defined by crystal symmetry. An approximate (isotropic) treatment of cell esds is used for estimating esds involving l.s. planes.

### Fractional atomic coordinates and isotropic or equivalent isotropic displacement parameters (Å^2^)

**Table:**

	*x*	*y*	*z*	*U*_iso_*/*U*_eq_	
O1	0.16345 (12)	0.76886 (14)	−0.02540 (14)	0.0871 (7)	
O2	0.09475 (10)	1.08899 (13)	0.43402 (13)	0.0703 (6)	
O3	0.19511 (12)	0.32014 (14)	0.35402 (16)	0.0890 (7)	
C1	0.09377 (16)	1.02145 (18)	0.37052 (19)	0.0568 (7)	
C2	0.03609 (16)	0.9860 (2)	0.3070 (2)	0.0666 (8)	
H2	−0.0069	1.0070	0.3045	0.080*	
C3	0.04228 (17)	0.91803 (19)	0.2459 (2)	0.0685 (8)	
H3	0.0030	0.8946	0.2026	0.082*	
C4	0.10415 (17)	0.88533 (19)	0.24821 (19)	0.0611 (8)	
C5	0.16111 (17)	0.9217 (2)	0.3127 (2)	0.0725 (9)	
H5	0.2039	0.9000	0.3156	0.087*	
C6	0.15690 (17)	0.9899 (2)	0.3739 (2)	0.0680 (8)	
H6	0.1963	1.0138	0.4165	0.082*	
C7	0.11115 (18)	0.81052 (19)	0.18306 (19)	0.0728 (9)	
H7	0.0646	0.7984	0.1416	0.087*	
C8	0.13470 (14)	0.72774 (17)	0.23821 (17)	0.0540 (7)	
H8A	0.1003	0.7096	0.2673	0.065*	
H8B	0.1763	0.7400	0.2901	0.065*	
C9	0.14816 (13)	0.65290 (17)	0.17931 (16)	0.0468 (7)	
C10	0.15636 (12)	0.56499 (17)	0.22096 (17)	0.0471 (7)	
C11	0.15574 (14)	0.48994 (19)	0.16688 (19)	0.0598 (8)	
H11	0.1469	0.4953	0.1006	0.072*	
C12	0.16766 (15)	0.4084 (2)	0.2074 (2)	0.0664 (8)	
H12	0.1670	0.3599	0.1687	0.080*	
C13	0.18067 (14)	0.39773 (19)	0.3056 (2)	0.0603 (8)	
C14	0.17958 (16)	0.4705 (2)	0.3607 (2)	0.0687 (9)	
H14	0.1870	0.4641	0.4266	0.082*	
C15	0.16775 (15)	0.55200 (19)	0.32023 (19)	0.0602 (8)	
H15	0.1672	0.5999	0.3593	0.072*	
C16	0.2115 (3)	0.2467 (2)	0.3082 (3)	0.1329 (17)	
H16A	0.2251	0.1997	0.3538	0.199*	
H16B	0.2484	0.2609	0.2834	0.199*	
H16C	0.1723	0.2293	0.2561	0.199*	
C17	0.03159 (16)	1.1250 (2)	0.4337 (2)	0.0766 (9)	
H17A	0.0054	1.0821	0.4564	0.092*	
H17B	0.0054	1.1422	0.3688	0.092*	
C18	0.04583 (16)	1.2034 (2)	0.4992 (2)	0.0624 (8)	
C19	0.01360 (17)	1.2129 (2)	0.5687 (2)	0.0813 (10)	
H19	−0.0156	1.1695	0.5775	0.098*	
C20	0.0249 (2)	1.2872 (3)	0.6252 (3)	0.1090 (13)	
H20	0.0027	1.2936	0.6716	0.131*	
C21	0.0673 (2)	1.3505 (3)	0.6144 (3)	0.1217 (16)	
H21	0.0743	1.4004	0.6528	0.146*	
C22	0.0995 (2)	1.3410 (2)	0.5475 (3)	0.1185 (15)	
H22	0.1292	1.3844	0.5400	0.142*	
C23	0.08895 (19)	1.2682 (2)	0.4904 (3)	0.0904 (11)	
H23	0.1117	1.2627	0.4445	0.108*	
C24	0.15546 (14)	0.66994 (19)	0.09195 (18)	0.0576 (8)	
H24	0.1613	0.6230	0.0547	0.069*	
C25	0.15466 (15)	0.7571 (2)	0.05331 (19)	0.0642 (8)	
C26	0.1498 (2)	0.8335 (2)	0.1167 (2)	0.0865 (11)	
H26	0.1968	0.8428	0.1585	0.104*	
C27	0.1309 (2)	0.9172 (2)	0.0609 (3)	0.1127 (14)	
H27A	0.1608	0.9264	0.0226	0.169*	
H27B	0.1351	0.9651	0.1050	0.169*	
H27C	0.0845	0.9136	0.0195	0.169*	
O1A	0.31086 (10)	0.78628 (13)	0.34094 (12)	0.0725 (6)	
O2A	0.41660 (10)	1.09227 (13)	0.87045 (14)	0.0687 (6)	
O3A	0.31787 (13)	0.31427 (14)	0.67545 (16)	0.0924 (8)	
C1A	0.41218 (15)	1.02388 (17)	0.80701 (18)	0.0520 (7)	
C2A	0.34719 (15)	0.99367 (19)	0.7637 (2)	0.0640 (8)	
H2A	0.3105	1.0188	0.7786	0.077*	
C3A	0.33616 (15)	0.9262 (2)	0.6981 (2)	0.0671 (8)	
H3A	0.2920	0.9055	0.6701	0.080*	
C4A	0.38969 (15)	0.88854 (17)	0.67276 (18)	0.0536 (7)	
C5A	0.45373 (15)	0.91928 (18)	0.71752 (19)	0.0581 (8)	
H5A	0.4905	0.8946	0.7023	0.070*	
C6A	0.46610 (15)	0.98615 (19)	0.78505 (19)	0.0610 (8)	
H6A	0.5105	1.0052	0.8152	0.073*	
C7A	0.37676 (15)	0.81644 (18)	0.59723 (18)	0.0614 (8)	
H7A	0.4214	0.8040	0.5892	0.074*	
C8A	0.35410 (14)	0.73180 (17)	0.63033 (17)	0.0563 (7)	
H8A1	0.3895	0.7118	0.6873	0.068*	
H8A2	0.3133	0.7427	0.6487	0.068*	
C9A	0.33916 (13)	0.66057 (17)	0.55616 (17)	0.0502 (7)	
C10A	0.33737 (13)	0.56976 (18)	0.58785 (17)	0.0501 (7)	
C11A	0.34401 (14)	0.4993 (2)	0.53198 (18)	0.0612 (8)	
H11A	0.3521	0.5102	0.4736	0.073*	
C12A	0.33929 (15)	0.4141 (2)	0.5583 (2)	0.0653 (8)	
H12A	0.3453	0.3687	0.5192	0.078*	
C13A	0.32555 (15)	0.39603 (19)	0.6431 (2)	0.0637 (8)	
C14A	0.31987 (18)	0.4637 (2)	0.7010 (2)	0.0805 (10)	
H14A	0.3116	0.4518	0.7590	0.097*	
C15A	0.32618 (15)	0.5493 (2)	0.67559 (19)	0.0678 (9)	
H15A	0.3230	0.5941	0.7171	0.081*	
C16A	0.3163 (2)	0.2418 (2)	0.6145 (3)	0.1197 (15)	
H16D	0.3133	0.1888	0.6482	0.180*	
H16E	0.3572	0.2410	0.5960	0.180*	
H16F	0.2772	0.2465	0.5579	0.180*	
C17A	0.48132 (16)	1.1279 (2)	0.9177 (2)	0.0749 (9)	
H17C	0.5054	1.1398	0.8713	0.090*	
H17D	0.5082	1.0866	0.9648	0.090*	
C18A	0.47207 (14)	1.21032 (18)	0.9665 (2)	0.0574 (8)	
C19A	0.50644 (16)	1.2251 (2)	1.0626 (2)	0.0757 (9)	
H19A	0.5340	1.1816	1.0994	0.091*	
C20A	0.5000 (2)	1.3042 (3)	1.1042 (2)	0.0982 (12)	
H20A	0.5234	1.3133	1.1691	0.118*	
C21A	0.4603 (2)	1.3691 (3)	1.0527 (3)	0.0980 (12)	
H21A	0.4567	1.4224	1.0815	0.118*	
C22A	0.42632 (18)	1.3549 (2)	0.9592 (3)	0.0905 (11)	
H22A	0.3984	1.3987	0.9234	0.109*	
C23A	0.43207 (16)	1.2772 (2)	0.9156 (2)	0.0741 (9)	
H23A	0.4085	1.2695	0.8505	0.089*	
C24A	0.32630 (14)	0.68225 (18)	0.46206 (18)	0.0550 (7)	
H24A	0.3189	0.6372	0.4171	0.066*	
C25A	0.32344 (14)	0.77137 (19)	0.42723 (18)	0.0560 (7)	
C26A	0.33199 (16)	0.84476 (18)	0.49948 (18)	0.0660 (8)	
H26A	0.2866	0.8546	0.5062	0.079*	
C27A	0.35178 (17)	0.92969 (18)	0.4628 (2)	0.0783 (10)	
H27D	0.3242	0.9385	0.3971	0.117*	
H27E	0.3991	0.9276	0.4662	0.117*	
H27F	0.3446	0.9771	0.5018	0.117*	

### Atomic displacement parameters (Å^2^)

**Table:**

	*U*^11^	*U*^22^	*U*^33^	*U*^12^	*U*^13^	*U*^23^
O1	0.137 (2)	0.0897 (17)	0.0539 (12)	0.0068 (13)	0.0580 (13)	0.0091 (11)
O2	0.0811 (15)	0.0689 (14)	0.0623 (12)	0.0179 (11)	0.0242 (10)	−0.0115 (11)
O3	0.142 (2)	0.0552 (15)	0.0934 (16)	0.0115 (13)	0.0706 (15)	0.0121 (13)
C1	0.078 (2)	0.0490 (19)	0.0469 (16)	0.0108 (16)	0.0249 (15)	0.0037 (15)
C2	0.077 (2)	0.064 (2)	0.0652 (19)	0.0079 (17)	0.0317 (17)	−0.0022 (17)
C3	0.085 (2)	0.063 (2)	0.0616 (18)	0.0008 (17)	0.0284 (16)	−0.0073 (17)
C4	0.089 (2)	0.0539 (19)	0.0407 (15)	0.0054 (18)	0.0209 (16)	0.0042 (14)
C5	0.090 (2)	0.069 (2)	0.0664 (19)	0.0242 (18)	0.0344 (18)	0.0026 (18)
C6	0.083 (2)	0.066 (2)	0.0554 (17)	0.0114 (18)	0.0213 (16)	−0.0032 (16)
C7	0.120 (3)	0.061 (2)	0.0453 (16)	0.0132 (18)	0.0367 (17)	0.0002 (15)
C8	0.0754 (19)	0.0525 (19)	0.0412 (14)	−0.0031 (14)	0.0284 (13)	−0.0042 (14)
C9	0.0563 (17)	0.0478 (18)	0.0386 (13)	−0.0010 (13)	0.0182 (12)	−0.0064 (13)
C10	0.0566 (17)	0.0483 (18)	0.0419 (13)	−0.0023 (13)	0.0231 (12)	−0.0076 (14)
C11	0.078 (2)	0.058 (2)	0.0452 (15)	−0.0028 (16)	0.0214 (14)	−0.0085 (16)
C12	0.085 (2)	0.057 (2)	0.0624 (18)	−0.0053 (16)	0.0308 (16)	−0.0175 (17)
C13	0.081 (2)	0.0457 (19)	0.0661 (18)	−0.0003 (15)	0.0404 (16)	0.0031 (17)
C14	0.108 (3)	0.059 (2)	0.0527 (17)	0.0027 (17)	0.0448 (17)	−0.0004 (17)
C15	0.091 (2)	0.0497 (19)	0.0504 (16)	0.0001 (15)	0.0378 (15)	−0.0039 (15)
C16	0.231 (5)	0.065 (3)	0.123 (3)	0.039 (3)	0.084 (3)	0.007 (3)
C17	0.082 (2)	0.074 (2)	0.077 (2)	0.0138 (18)	0.0280 (17)	−0.0069 (18)
C18	0.081 (2)	0.058 (2)	0.0554 (17)	0.0123 (17)	0.0311 (15)	0.0003 (16)
C19	0.099 (3)	0.086 (3)	0.072 (2)	−0.008 (2)	0.0455 (19)	−0.002 (2)
C20	0.136 (4)	0.126 (4)	0.094 (3)	−0.013 (3)	0.078 (3)	−0.041 (3)
C21	0.169 (4)	0.085 (3)	0.143 (4)	−0.024 (3)	0.096 (4)	−0.049 (3)
C22	0.182 (4)	0.064 (3)	0.150 (4)	−0.022 (3)	0.109 (3)	−0.027 (3)
C23	0.133 (3)	0.071 (3)	0.096 (3)	−0.004 (2)	0.079 (2)	−0.005 (2)
C24	0.077 (2)	0.060 (2)	0.0412 (14)	0.0015 (15)	0.0271 (13)	−0.0041 (14)
C25	0.087 (2)	0.069 (2)	0.0443 (15)	0.0024 (16)	0.0328 (15)	0.0007 (16)
C26	0.156 (3)	0.057 (2)	0.072 (2)	−0.003 (2)	0.072 (2)	−0.0001 (18)
C27	0.215 (5)	0.065 (2)	0.087 (2)	0.015 (3)	0.088 (3)	0.014 (2)
O1A	0.1121 (17)	0.0722 (14)	0.0350 (10)	−0.0027 (11)	0.0252 (10)	0.0048 (10)
O2A	0.0722 (14)	0.0668 (14)	0.0740 (13)	−0.0165 (11)	0.0329 (10)	−0.0276 (11)
O3A	0.160 (2)	0.0531 (15)	0.0759 (15)	0.0002 (13)	0.0538 (15)	0.0073 (13)
C1A	0.072 (2)	0.0428 (17)	0.0463 (15)	−0.0049 (15)	0.0251 (14)	−0.0036 (13)
C2A	0.061 (2)	0.074 (2)	0.0643 (18)	−0.0085 (16)	0.0289 (15)	−0.0163 (17)
C3A	0.069 (2)	0.075 (2)	0.0588 (17)	−0.0219 (17)	0.0224 (15)	−0.0186 (17)
C4A	0.073 (2)	0.0475 (18)	0.0463 (15)	−0.0014 (16)	0.0273 (15)	−0.0013 (14)
C5A	0.067 (2)	0.0558 (19)	0.0552 (16)	0.0015 (15)	0.0241 (15)	−0.0093 (15)
C6A	0.0602 (19)	0.064 (2)	0.0586 (17)	−0.0049 (16)	0.0182 (15)	−0.0082 (16)
C7A	0.085 (2)	0.056 (2)	0.0453 (15)	−0.0053 (16)	0.0231 (14)	−0.0045 (14)
C8A	0.080 (2)	0.0552 (19)	0.0351 (14)	0.0022 (15)	0.0189 (13)	−0.0016 (13)
C9A	0.0621 (18)	0.0490 (18)	0.0422 (14)	0.0017 (13)	0.0200 (12)	−0.0006 (13)
C10A	0.0636 (18)	0.0516 (19)	0.0366 (13)	0.0010 (14)	0.0177 (12)	−0.0045 (14)
C11A	0.086 (2)	0.059 (2)	0.0448 (15)	−0.0015 (16)	0.0300 (15)	0.0005 (15)
C12A	0.094 (2)	0.051 (2)	0.0571 (17)	−0.0006 (16)	0.0315 (15)	−0.0081 (15)
C13A	0.095 (2)	0.048 (2)	0.0507 (17)	0.0035 (16)	0.0271 (16)	0.0075 (16)
C14A	0.139 (3)	0.066 (2)	0.0494 (17)	0.011 (2)	0.0480 (19)	0.0085 (17)
C15A	0.108 (3)	0.058 (2)	0.0445 (16)	0.0095 (17)	0.0330 (16)	0.0047 (15)
C16A	0.203 (5)	0.060 (3)	0.118 (3)	−0.006 (3)	0.082 (3)	−0.006 (2)
C17A	0.075 (2)	0.066 (2)	0.083 (2)	−0.0095 (18)	0.0219 (17)	−0.0221 (18)
C18A	0.069 (2)	0.0463 (18)	0.0576 (17)	−0.0063 (15)	0.0204 (15)	−0.0087 (16)
C19A	0.087 (2)	0.077 (3)	0.0570 (19)	0.0107 (18)	0.0135 (17)	0.0019 (18)
C20A	0.121 (3)	0.108 (3)	0.0535 (19)	0.014 (3)	0.009 (2)	−0.028 (2)
C21A	0.118 (3)	0.080 (3)	0.084 (3)	0.008 (2)	0.013 (2)	−0.032 (2)
C22A	0.116 (3)	0.060 (2)	0.085 (2)	0.022 (2)	0.016 (2)	−0.006 (2)
C23A	0.094 (2)	0.069 (2)	0.0540 (18)	0.0051 (19)	0.0150 (17)	−0.0060 (18)
C24A	0.076 (2)	0.0519 (19)	0.0397 (14)	−0.0051 (14)	0.0214 (13)	−0.0058 (14)
C25A	0.0711 (19)	0.059 (2)	0.0409 (15)	−0.0023 (15)	0.0217 (13)	0.0021 (14)
C26A	0.099 (2)	0.058 (2)	0.0440 (15)	−0.0051 (16)	0.0271 (15)	−0.0009 (15)
C27A	0.122 (3)	0.060 (2)	0.0555 (17)	−0.0166 (18)	0.0302 (18)	0.0059 (16)

### Geometric parameters (Å, º)

**Table:**

O1—C25	1.228 (3)	O1A—C25A	1.224 (3)
O2—C1	1.382 (3)	O2A—C1A	1.380 (3)
O2—C17	1.410 (3)	O2A—C17A	1.406 (3)
O3—C13	1.366 (3)	O3A—C13A	1.362 (3)
O3—C16	1.399 (4)	O3A—C16A	1.414 (4)
C1—C6	1.371 (4)	C1A—C2A	1.371 (4)
C1—C2	1.372 (4)	C1A—C6A	1.372 (4)
C2—C3	1.399 (4)	C2A—C3A	1.376 (4)
C2—H2	0.9300	C2A—H2A	0.9300
C3—C4	1.358 (4)	C3A—C4A	1.390 (4)
C3—H3	0.9300	C3A—H3A	0.9300
C4—C5	1.373 (4)	C4A—C5A	1.361 (4)
C4—C7	1.521 (4)	C4A—C7A	1.522 (4)
C5—C6	1.392 (4)	C5A—C6A	1.387 (4)
C5—H5	0.9300	C5A—H5A	0.9300
C6—H6	0.9300	C6A—H6A	0.9300
C7—C26	1.472 (4)	C7A—C26A	1.502 (4)
C7—C8	1.497 (4)	C7A—C8A	1.504 (4)
C7—H7	0.9800	C7A—H7A	0.9800
C8—C9	1.506 (3)	C8A—C9A	1.498 (3)
C8—H8A	0.9700	C8A—H8A1	0.9700
C8—H8B	0.9700	C8A—H8A2	0.9700
C9—C24	1.352 (3)	C9A—C24A	1.355 (3)
C9—C10	1.463 (3)	C9A—C10A	1.467 (3)
C10—C11	1.390 (3)	C10A—C11A	1.383 (3)
C10—C15	1.406 (3)	C10A—C15A	1.402 (3)
C11—C12	1.369 (4)	C11A—C12A	1.369 (4)
C11—H11	0.9300	C11A—H11A	0.9300
C12—C13	1.380 (4)	C12A—C13A	1.377 (4)
C12—H12	0.9300	C12A—H12A	0.9300
C13—C14	1.376 (4)	C13A—C14A	1.362 (4)
C14—C15	1.368 (4)	C14A—C15A	1.378 (4)
C14—H14	0.9300	C14A—H14A	0.9300
C15—H15	0.9300	C15A—H15A	0.9300
C16—H16A	0.9600	C16A—H16D	0.9600
C16—H16B	0.9600	C16A—H16E	0.9600
C16—H16C	0.9600	C16A—H16F	0.9600
C17—C18	1.503 (4)	C17A—C18A	1.488 (4)
C17—H17A	0.9700	C17A—H17C	0.9700
C17—H17B	0.9700	C17A—H17D	0.9700
C18—C23	1.362 (4)	C18A—C23A	1.377 (4)
C18—C19	1.378 (4)	C18A—C19A	1.379 (4)
C19—C20	1.380 (4)	C19A—C20A	1.377 (4)
C19—H19	0.9300	C19A—H19A	0.9300
C20—C21	1.344 (5)	C20A—C21A	1.355 (5)
C20—H20	0.9300	C20A—H20A	0.9300
C21—C22	1.345 (5)	C21A—C22A	1.344 (4)
C21—H21	0.9300	C21A—H21A	0.9300
C22—C23	1.367 (4)	C22A—C23A	1.369 (4)
C22—H22	0.9300	C22A—H22A	0.9300
C23—H23	0.9300	C23A—H23A	0.9300
C24—C25	1.445 (4)	C24A—C25A	1.449 (4)
C24—H24	0.9300	C24A—H24A	0.9300
C25—C26	1.510 (4)	C25A—C26A	1.511 (4)
C26—C27	1.502 (4)	C26A—C27A	1.507 (4)
C26—H26	0.9800	C26A—H26A	0.9800
C27—H27A	0.9600	C27A—H27D	0.9600
C27—H27B	0.9600	C27A—H27E	0.9600
C27—H27C	0.9600	C27A—H27F	0.9600
			
C1—O2—C17	117.8 (2)	C1A—O2A—C17A	118.6 (2)
C13—O3—C16	119.7 (2)	C13A—O3A—C16A	119.0 (2)
C6—C1—C2	119.9 (3)	C2A—C1A—C6A	119.6 (3)
C6—C1—O2	114.8 (3)	C2A—C1A—O2A	114.7 (2)
C2—C1—O2	125.3 (3)	C6A—C1A—O2A	125.7 (3)
C1—C2—C3	119.6 (3)	C1A—C2A—C3A	120.1 (3)
C1—C2—H2	120.2	C1A—C2A—H2A	120.0
C3—C2—H2	120.2	C3A—C2A—H2A	120.0
C4—C3—C2	121.7 (3)	C2A—C3A—C4A	121.4 (3)
C4—C3—H3	119.1	C2A—C3A—H3A	119.3
C2—C3—H3	119.1	C4A—C3A—H3A	119.3
C3—C4—C5	117.6 (3)	C5A—C4A—C3A	117.3 (3)
C3—C4—C7	122.0 (3)	C5A—C4A—C7A	121.6 (3)
C5—C4—C7	120.4 (3)	C3A—C4A—C7A	121.1 (3)
C4—C5—C6	122.2 (3)	C4A—C5A—C6A	122.2 (3)
C4—C5—H5	118.9	C4A—C5A—H5A	118.9
C6—C5—H5	118.9	C6A—C5A—H5A	118.9
C1—C6—C5	119.0 (3)	C1A—C6A—C5A	119.4 (3)
C1—C6—H6	120.5	C1A—C6A—H6A	120.3
C5—C6—H6	120.5	C5A—C6A—H6A	120.3
C26—C7—C8	113.8 (3)	C26A—C7A—C8A	113.1 (2)
C26—C7—C4	113.9 (3)	C26A—C7A—C4A	113.4 (2)
C8—C7—C4	112.2 (2)	C8A—C7A—C4A	113.2 (2)
C26—C7—H7	105.3	C26A—C7A—H7A	105.4
C8—C7—H7	105.3	C8A—C7A—H7A	105.4
C4—C7—H7	105.3	C4A—C7A—H7A	105.4
C7—C8—C9	115.0 (2)	C9A—C8A—C7A	114.1 (2)
C7—C8—H8A	108.5	C9A—C8A—H8A1	108.7
C9—C8—H8A	108.5	C7A—C8A—H8A1	108.7
C7—C8—H8B	108.5	C9A—C8A—H8A2	108.7
C9—C8—H8B	108.5	C7A—C8A—H8A2	108.7
H8A—C8—H8B	107.5	H8A1—C8A—H8A2	107.6
C24—C9—C10	122.4 (2)	C24A—C9A—C10A	122.3 (2)
C24—C9—C8	118.9 (2)	C24A—C9A—C8A	119.0 (2)
C10—C9—C8	118.7 (2)	C10A—C9A—C8A	118.7 (2)
C11—C10—C15	115.9 (2)	C11A—C10A—C15A	115.9 (2)
C11—C10—C9	122.9 (2)	C11A—C10A—C9A	122.4 (2)
C15—C10—C9	121.2 (2)	C15A—C10A—C9A	121.7 (2)
C12—C11—C10	122.5 (3)	C12A—C11A—C10A	123.2 (2)
C12—C11—H11	118.7	C12A—C11A—H11A	118.4
C10—C11—H11	118.7	C10A—C11A—H11A	118.4
C11—C12—C13	120.4 (3)	C11A—C12A—C13A	119.6 (3)
C11—C12—H12	119.8	C11A—C12A—H12A	120.2
C13—C12—H12	119.8	C13A—C12A—H12A	120.2
O3—C13—C14	116.1 (3)	O3A—C13A—C14A	116.2 (3)
O3—C13—C12	125.5 (3)	O3A—C13A—C12A	124.9 (3)
C14—C13—C12	118.4 (3)	C14A—C13A—C12A	118.9 (3)
C15—C14—C13	121.1 (3)	C13A—C14A—C15A	121.5 (3)
C15—C14—H14	119.4	C13A—C14A—H14A	119.3
C13—C14—H14	119.4	C15A—C14A—H14A	119.3
C14—C15—C10	121.5 (3)	C14A—C15A—C10A	120.8 (3)
C14—C15—H15	119.2	C14A—C15A—H15A	119.6
C10—C15—H15	119.2	C10A—C15A—H15A	119.6
O3—C16—H16A	109.5	O3A—C16A—H16D	109.5
O3—C16—H16B	109.5	O3A—C16A—H16E	109.5
H16A—C16—H16B	109.5	H16D—C16A—H16E	109.5
O3—C16—H16C	109.5	O3A—C16A—H16F	109.5
H16A—C16—H16C	109.5	H16D—C16A—H16F	109.5
H16B—C16—H16C	109.5	H16E—C16A—H16F	109.5
O2—C17—C18	108.1 (3)	O2A—C17A—C18A	108.7 (2)
O2—C17—H17A	110.1	O2A—C17A—H17C	110.0
C18—C17—H17A	110.1	C18A—C17A—H17C	110.0
O2—C17—H17B	110.1	O2A—C17A—H17D	110.0
C18—C17—H17B	110.1	C18A—C17A—H17D	110.0
H17A—C17—H17B	108.4	H17C—C17A—H17D	108.3
C23—C18—C19	117.8 (3)	C23A—C18A—C19A	117.3 (3)
C23—C18—C17	121.7 (3)	C23A—C18A—C17A	121.0 (3)
C19—C18—C17	120.5 (3)	C19A—C18A—C17A	121.5 (3)
C18—C19—C20	119.7 (3)	C20A—C19A—C18A	120.1 (3)
C18—C19—H19	120.2	C20A—C19A—H19A	119.9
C20—C19—H19	120.2	C18A—C19A—H19A	119.9
C21—C20—C19	121.3 (3)	C21A—C20A—C19A	121.5 (3)
C21—C20—H20	119.4	C21A—C20A—H20A	119.2
C19—C20—H20	119.4	C19A—C20A—H20A	119.2
C20—C21—C22	119.3 (4)	C22A—C21A—C20A	118.7 (3)
C20—C21—H21	120.4	C22A—C21A—H21A	120.7
C22—C21—H21	120.4	C20A—C21A—H21A	120.7
C21—C22—C23	120.6 (4)	C21A—C22A—C23A	121.2 (3)
C21—C22—H22	119.7	C21A—C22A—H22A	119.4
C23—C22—H22	119.7	C23A—C22A—H22A	119.4
C18—C23—C22	121.4 (3)	C22A—C23A—C18A	121.2 (3)
C18—C23—H23	119.3	C22A—C23A—H23A	119.4
C22—C23—H23	119.3	C18A—C23A—H23A	119.4
C9—C24—C25	123.6 (3)	C9A—C24A—C25A	124.0 (2)
C9—C24—H24	118.2	C9A—C24A—H24A	118.0
C25—C24—H24	118.2	C25A—C24A—H24A	118.0
O1—C25—C24	120.8 (3)	O1A—C25A—C24A	120.5 (2)
O1—C25—C26	120.9 (3)	O1A—C25A—C26A	121.3 (3)
C24—C25—C26	118.0 (2)	C24A—C25A—C26A	118.1 (2)
C7—C26—C27	117.1 (3)	C7A—C26A—C27A	115.5 (2)
C7—C26—C25	111.6 (3)	C7A—C26A—C25A	110.6 (2)
C27—C26—C25	112.6 (2)	C27A—C26A—C25A	112.3 (2)
C7—C26—H26	104.8	C7A—C26A—H26A	105.9
C27—C26—H26	104.8	C27A—C26A—H26A	105.9
C25—C26—H26	104.8	C25A—C26A—H26A	105.9
C26—C27—H27A	109.5	C26A—C27A—H27D	109.5
C26—C27—H27B	109.5	C26A—C27A—H27E	109.5
H27A—C27—H27B	109.5	H27D—C27A—H27E	109.5
C26—C27—H27C	109.5	C26A—C27A—H27F	109.5
H27A—C27—H27C	109.5	H27D—C27A—H27F	109.5
H27B—C27—H27C	109.5	H27E—C27A—H27F	109.5
			
C17—O2—C1—C6	179.5 (2)	C17A—O2A—C1A—C2A	−179.5 (2)
C17—O2—C1—C2	−0.2 (4)	C17A—O2A—C1A—C6A	0.1 (4)
C6—C1—C2—C3	−0.2 (4)	C6A—C1A—C2A—C3A	−0.4 (4)
O2—C1—C2—C3	179.5 (2)	O2A—C1A—C2A—C3A	179.2 (2)
C1—C2—C3—C4	0.6 (4)	C1A—C2A—C3A—C4A	−1.3 (4)
C2—C3—C4—C5	−0.3 (4)	C2A—C3A—C4A—C5A	1.9 (4)
C2—C3—C4—C7	179.1 (2)	C2A—C3A—C4A—C7A	−177.6 (2)
C3—C4—C5—C6	−0.3 (4)	C3A—C4A—C5A—C6A	−0.7 (4)
C7—C4—C5—C6	−179.7 (2)	C7A—C4A—C5A—C6A	178.7 (2)
C2—C1—C6—C5	−0.4 (4)	C2A—C1A—C6A—C5A	1.5 (4)
O2—C1—C6—C5	179.9 (2)	O2A—C1A—C6A—C5A	−178.1 (2)
C4—C5—C6—C1	0.7 (4)	C4A—C5A—C6A—C1A	−0.9 (4)
C3—C4—C7—C26	118.8 (3)	C5A—C4A—C7A—C26A	−118.0 (3)
C5—C4—C7—C26	−61.8 (4)	C3A—C4A—C7A—C26A	61.4 (4)
C3—C4—C7—C8	−110.1 (3)	C5A—C4A—C7A—C8A	111.5 (3)
C5—C4—C7—C8	69.2 (4)	C3A—C4A—C7A—C8A	−69.1 (3)
C26—C7—C8—C9	−44.2 (4)	C26A—C7A—C8A—C9A	47.7 (3)
C4—C7—C8—C9	−175.3 (2)	C4A—C7A—C8A—C9A	178.4 (2)
C7—C8—C9—C24	16.6 (4)	C7A—C8A—C9A—C24A	−20.0 (4)
C7—C8—C9—C10	−166.5 (2)	C7A—C8A—C9A—C10A	161.4 (2)
C24—C9—C10—C11	−16.0 (4)	C24A—C9A—C10A—C11A	20.8 (4)
C8—C9—C10—C11	167.3 (2)	C8A—C9A—C10A—C11A	−160.6 (2)
C24—C9—C10—C15	161.8 (3)	C24A—C9A—C10A—C15A	−156.7 (3)
C8—C9—C10—C15	−14.9 (4)	C8A—C9A—C10A—C15A	21.8 (4)
C15—C10—C11—C12	−2.1 (4)	C15A—C10A—C11A—C12A	0.8 (4)
C9—C10—C11—C12	175.9 (3)	C9A—C10A—C11A—C12A	−176.8 (3)
C10—C11—C12—C13	0.3 (4)	C10A—C11A—C12A—C13A	1.7 (4)
C16—O3—C13—C14	−166.5 (3)	C16A—O3A—C13A—C14A	173.7 (3)
C16—O3—C13—C12	13.2 (5)	C16A—O3A—C13A—C12A	−7.3 (5)
C11—C12—C13—O3	−178.0 (3)	C11A—C12A—C13A—O3A	178.2 (3)
C11—C12—C13—C14	1.7 (4)	C11A—C12A—C13A—C14A	−2.8 (4)
O3—C13—C14—C15	178.0 (3)	O3A—C13A—C14A—C15A	−179.5 (3)
C12—C13—C14—C15	−1.8 (4)	C12A—C13A—C14A—C15A	1.4 (5)
C13—C14—C15—C10	−0.1 (4)	C13A—C14A—C15A—C10A	1.3 (5)
C11—C10—C15—C14	2.0 (4)	C11A—C10A—C15A—C14A	−2.3 (4)
C9—C10—C15—C14	−176.0 (3)	C9A—C10A—C15A—C14A	175.4 (3)
C1—O2—C17—C18	−173.3 (2)	C1A—O2A—C17A—C18A	169.1 (2)
O2—C17—C18—C23	51.4 (4)	O2A—C17A—C18A—C23A	−55.5 (4)
O2—C17—C18—C19	−130.6 (3)	O2A—C17A—C18A—C19A	128.7 (3)
C23—C18—C19—C20	1.1 (5)	C23A—C18A—C19A—C20A	0.1 (5)
C17—C18—C19—C20	−177.0 (3)	C17A—C18A—C19A—C20A	176.1 (3)
C18—C19—C20—C21	−0.6 (6)	C18A—C19A—C20A—C21A	−0.1 (6)
C19—C20—C21—C22	−0.2 (7)	C19A—C20A—C21A—C22A	0.6 (6)
C20—C21—C22—C23	0.5 (7)	C20A—C21A—C22A—C23A	−1.0 (6)
C19—C18—C23—C22	−0.8 (5)	C21A—C22A—C23A—C18A	1.0 (6)
C17—C18—C23—C22	177.2 (3)	C19A—C18A—C23A—C22A	−0.5 (5)
C21—C22—C23—C18	0.1 (7)	C17A—C18A—C23A—C22A	−176.5 (3)
C10—C9—C24—C25	−173.4 (2)	C10A—C9A—C24A—C25A	176.1 (2)
C8—C9—C24—C25	3.4 (4)	C8A—C9A—C24A—C25A	−2.4 (4)
C9—C24—C25—O1	177.9 (3)	C9A—C24A—C25A—O1A	−179.3 (3)
C9—C24—C25—C26	4.0 (4)	C9A—C24A—C25A—C26A	−3.0 (4)
C8—C7—C26—C27	−178.1 (3)	C8A—C7A—C26A—C27A	179.4 (2)
C4—C7—C26—C27	−47.8 (4)	C4A—C7A—C26A—C27A	48.9 (4)
C8—C7—C26—C25	50.2 (4)	C8A—C7A—C26A—C25A	−51.6 (3)
C4—C7—C26—C25	−179.5 (3)	C4A—C7A—C26A—C25A	177.8 (2)
O1—C25—C26—C7	155.3 (3)	O1A—C25A—C26A—C7A	−153.9 (3)
C24—C25—C26—C7	−30.8 (4)	C24A—C25A—C26A—C7A	29.9 (4)
O1—C25—C26—C27	21.3 (5)	O1A—C25A—C26A—C27A	−23.3 (4)
C24—C25—C26—C27	−164.8 (3)	C24A—C25A—C26A—C27A	160.5 (3)

### Hydrogen-bond geometry (Å, º)

Cg3 and Cg8 are the centroids of rings C10-C15 and C10A-C15A, respectively.

**Table:**

*D*—H···*A*	*D*—H	H···*A*	*D*···*A*	*D*—H···*A*
C2*A*—H2*A*···*Cg*3^i^	0.93	2.87	3.707 (3)	150
C16*A*—H16*E*···*Cg*8^ii^	0.96	2.92	3.866 (5)	168

Symmetry codes: (i) *x*, −*y*+3/2, *z*+1/2; (ii) *x*, −*y*+3/2, *z*−1/2.
